# Age-specific breast and ovarian cancer risks associated with germline *BRCA1* or *BRCA2* pathogenic variants – an Asian study of 572 families

**DOI:** 10.1016/j.lanwpc.2024.101017

**Published:** 2024-02-05

**Authors:** Weang-Kee Ho, Nur Tiara Hassan, Sook-Yee Yoon, Xin Yang, Joanna M.C. Lim, Nur Diana Binte Ishak, Peh Joo Ho, Eldarina A. Wijaya, Patsy Pei-Sze Ng, Craig Luccarini, Jamie Allen, Mei-Chee Tai, Jianbang Chiang, Zewen Zhang, Mee-Hoong See, Meow-Keong Thong, Yin-Ling Woo, Alison M. Dunning, Mikael Hartman, Cheng-Har Yip, Nur Aishah Mohd Taib, Douglas F. Easton, Jingmei Li, Joanne Ngeow, Antonis C. Antoniou, Soo-Hwang Teo

**Affiliations:** a School of Mathematical Sciences, Faculty of Science and Engineering, University of Nottingham Malaysia, Jalan Broga, Semenyih, 43500, Selangor, Malaysia; b Cancer Research Malaysia, 1 Jalan SS12/1A, Subang Jaya, 47500, Selangor, Malaysia; c Centre for Cancer Genetic Epidemiology, Department of Public Health and Primary Care, University of Cambridge, CB1 8RN, Cambridge, UK; d Cancer Genetics Service, National Cancer Centre Singapore, Singapore; e Department of Surgery, Yong Loo Lin School of Medicine, National University of Singapore and National University Health System, Singapore; f Saw Swee Hock School of Public Health, National University of Singapore and National University Health System, Singapore, Singapore; g Genome Institute of Singapore (GIS), Agency for Science, Technology and Research (A∗STAR), 60 Biopolis Street, Genome, #02-01, Singapore, 138672, Singapore; h Centre for Cancer Genetic Epidemiology, Department of Oncology, University of Cambridge, 2 Worts' Causeway, CB1 8RN, Cambridge, UK; i European Molecular Biology Laboratory, European Bioinformatics Institute, Wellcome Genome Campus, UK; j Department of Surgery, Faculty of Medicine, University of Malaya, Jalan Universiti, Kuala Lumpur, 50630, Malaysia; k Genetic Medicine Unit, University of Malaya Medical Center, Kuala Lumpur, Malaysia; l Department of Obstetrics and Gynaecology, Faculty of Medicine, University of Malaya, Kuala Lumpur, Malaysia; m Department of Surgery, National University Hospital and National University Health System, Singapore, Singapore; n Subang Jaya Medical Centre, 1 Jalan SS12/1A, Subang Jaya, 47500, Selangor, Malaysia; o University Malaya Cancer Research Institute, 50603, Kuala Lumpur, Malaysia; p Lee Kong Chian School of Medicine, Nanyang Technological University, Singapore, Singapore

**Keywords:** Penetrance, Breast cancer risk, Ovarian cancer risk, BRCA1, BRCA2

## Abstract

**Background:**

Clinical management of Asian *BRCA1* and *BRCA2* pathogenic variants (PV) carriers remains challenging due to imprecise age-specific breast (BC) and ovarian cancer (OC) risks estimates. We aimed to refine these estimates using six multi-ethnic studies in Asia.

**Methods:**

Data were collected on 271 *BRCA1* and 301 *BRCA2* families from Malaysia and Singapore, ascertained through population/hospital-based case-series (88%) and genetic clinics (12%). Age-specific cancer risks were estimated using a modified segregation analysis method, adjusted for ascertainment.

**Findings:**

BC and OC relative risks (RRs) varied across age groups for both *BRCA1* and *BRCA*2. The age-specific RR estimates were similar across ethnicities and country of residence. For *BRCA1* carriers of Malay, Indian and Chinese ancestry born between 1950 and 1959 in Malaysia, the cumulative risk (95% CI) of BC by age 80 was 40% (36%–44%), 49% (44%–53%) and 55% (51%–60%), respectively. The corresponding estimates for *BRCA2* were 29% (26–32%), 36% (33%–40%) and 42% (38%–45%). The corresponding cumulative BC risks for Singapore residents from the same birth cohort, where the underlying population cancer incidences are higher compared to Malaysia, were higher, varying by ancestry group between 57 and 61% for *BRCA1,* and between 43 and 47% for *BRCA2* carriers. The cumulative risk of OC by age 80 was 31% (27–36%) for *BRCA1* and 12% (10%–15%) for *BRCA2* carriers in Malaysia born between 1950 and 1959; and 42% (34–50%) for *BRCA1* and 20% (14–27%) for *BRCA2* carriers of the same birth cohort in Singapore. There was evidence of increased BC and OC risks for women from >1960 birth cohorts (p-value = 3.6 × 10^−5^ for *BRCA1* and 0.018 for *BRCA*2).

**Interpretation:**

The absolute age-specific cancer risks of Asian carriers vary depending on the underlying population-specific cancer incidences, and hence should be customised to allow for more accurate cancer risk management.

**Funding:**

Wellcome Trust [grant no: v203477/Z/16/Z]; CRUK (PPRPGM-Nov20∖100002).

**Research in context**

**Evidence before this study**

To date, four studies have reported breast cancer risk estimates for *BRCA1* and *BRCA2* pathogenic variant carriers of Asian ancestry, of which two also reported the risk of ovarian cancer. These studies were based on small numbers of pathogenic variant carriers and there is considerable variability in the relative risk estimates between these studies. Moreover, all the published Asian studies assumed a constant relative risk across all ages, which has been shown in European studies to result in imprecise age specific absolute risks. Taken together, there is a need to provide more accurate lifetime and age-specific risk estimates for Asian BRCA carriers, both for the purposes of management of lifetime risk of cancers, and also for informing at what age risk-reducing are required.

**Added value of this study**

We have refined age-specific breast and ovarian cancer risks for *BRCA1* and *BRCA2* carriers of Chinese ancestry and quantified for the first time the cancer risks for carriers of Malay and Indian ancestry. Our findings show that the cancer relative risks in Asian carriers, vary by age, highlighting the importance of using age-specific relative risks for estimating absolute risks of breast and ovarian cancer. Furthermore, the age-specific relative risk estimates were similar across the three ethnic groups and close with those reported for European carriers. Despite similarities in age-specific relative risks, there were marked differences between the absolute risks for Asian carriers of all ancestries residing in Malaysia, a developing Asian country, and the absolute risks for European carriers. However, the absolute risks for Asian carriers residing in Singapore, a developed Asian country, were similar to those for European carriers.

**Implications of all the available evidence**

The findings provide a framework for estimating age-specific absolute risks for Asian carriers of diverse ancestries and place of residence. Given the comparable relative risk estimates among Asian ethnic subgroups, the absolute age-specific cancer risks for Asian carriers will differ, and will depend on the underlying population-specific incidences. As a result, a clinical management strategy based on European age-specific cancer risks may be suitable for Asian carriers residing in countries with population cancer incidences similar to high income Asian countries like Singapore. However, for Asian carriers residing in countries with markedly lower population cancer incidences, as in Malaysia, it is expected that their age-specific cancer risks would be lower than the risks applicable for European ancestry carriers. The results will optimize the clinical management of Asian *BRCA1* and *BRCA2* pathogenic variant carriers from diverse ancestries in Asia and beyond.

## Introduction

Female carriers of *BRCA1* or *BRCA2* pathogenic variants (PV) have increased risks of both breast and ovarian cancer that warrant enhanced surveillance or risk-reducing options.^1^, ^2^, ^3^, ^4^, ^5^ Large-scale prospective cohort of primarily high-risk European-ancestry carriers have estimated that the breast cancer cumulative risk to age 70 was 66% (95% CI: 61–72%) for *BRCA1* and 61% (95% CI: 55–68%) for *BRCA2* and the corresponding ovarian cancer cumulative risks were 41% (95% CI: 33–50%) and 15% (95% CI: 10–23%), respectively.^6^ Notably cancer risk estimates for carriers of European ancestry have been found to vary by age, birth cohort and mutation position.^1^^,^^4^, ^5^, ^6^, ^7^, ^8^ The estimates from these studies have formed the basis for developing age-specific clinical management guidelines on screening and prophylactic surgery for European-ancestry PV carriers.

By contrast, only four studies (eTable 1) have reported breast cancer risk estimates for Asian carriers of *BRCA1* and *BRCA2* PV, of which two also estimated ovarian cancer risks.^9^, ^10^, ^11^, ^12^ These cumulative cancer risk estimates are imprecise and there was substantial variability in the relative risk estimates between studies, likely due to differences in sampling and analytical methods. Moreover, all existing Asian studies, due to the small sizes (125–376 *BRCA1* and *BRCA2* families), assumed a constant relative risk across all ages and birth cohorts, which is sub-optimal for informing risk-reducing clinical decisions which depend critically on age. Taken together, based on paucity of accurate Asian-specific data, clinical management of Asian PV carriers remains challenging.

Here, we use data from 572 families from Malaysia and Singapore with *BRCA1* and *BRCA2* PVs to estimate age-specific relative and absolute breast and ovarian cancer risks. We also investigate how these risks vary by ethnicity, birth cohort and PV location.

## Methods

### Families

Data were obtained on families in Malaysia and Singapore in which a *BRCA1* or *BRCA2* PV was present.^13^, ^14^, ^15^, ^16^ Two ascertainment schemes were used: (1) four studies identified PVs by systematic screening of cancer case-series, unselected for family history (235 *BRCA1* and 269 *BRCA2* families), and (2) two studies screened breast and ovarian cancer patients from multiple-case families recruited through genetics clinics (36 *BRCA1* and 32 *BRCA2* families). Patients recruited through the case-series and the first family member recruited through multiple-case families was designated as the proband. Genetic testing was offered to family members of probands. Variants were considered pathogenic or likely pathogenic based on ACMG/AMP guidelines.^17^ The number of eligible families by gene and recruitment scheme are detailed in eTable 2. Details regarding study recruitment, sequencing and PV definitions are provided in Supplementary Methods.

All studies were approved by the relevant institutional ethics committees and review boards, and all participants provided written informed consent for the study and for genetic testing.

### Statistical methods

The breast and ovarian cancer-specific relative risks (RRs) were estimated simultaneously, but separately, for *BRCA1* and *BRCA2*, using modified segregation analysis.^18^ Pedigree likelihoods were constructed using first-degree family members of probands and maximised using pedigree analysis software MENDEL.^19^ Family members were censored at the age at first cancer diagnosis, age at death, age at last follow-up or age 80 years, whichever occurred first. Since ascertainment criteria varied across studies, ascertainment bias was adjusted for each family separately using the ascertainment assumption free method described and applied previously^1^^,^^4^^,^^20^ which has been shown to provide unbiased parameter estimates.^21^, ^22^, ^23^ Briefly, the likelihoods were computed conditional on any data that may be relevant to the ascertainment. For families recruited through population and hospital-based case-series, we maximised the conditional likelihood of the pedigree given the phenotypic and genotypic information of the proband. For families recruited through multiple-case families, we maximised the conditional likelihood of the pedigree given the genotypic information of the proband and phenotypic information of all family members. Mutation status of tested family members were included in the analyses.

We considered models in which: (1) the log RR was assumed to be constant across ages; and (2) the log RRs were assumed to be constant within 10-year age intervals (20–29, 30–39, 40–49, 50–59, 60–69, 70–79). We fitted the above models by constraining the overall cancer incidence over carriers and non-carriers in the model to agree with country-, ethnic- and birth cohort-specific population age-specific incidences.^24^ We also considered country-, cohort-, self-reported ethnicity and PV location-specific models, where an additional variable for each subgroup (assumed to be constant over all ages) was included in the model to allow for log RRs to differ between subgroups. Nested models were compared against each other using the likelihood ratio test (LRT). Details regarding the statistical analysis are provided in Supplementary Methods.

### Role of the funding sources

This study was supported by Wellcome Trust grant [grant no: v203477/Z/16/Z], which was not involved in any aspect of the study, including data collection, analysis, interpretation, trial design, patient recruitment, or any other pertinent aspect related to the writing of the manuscript or the decision to submit it for publication. The authors have not received any payment from pharmaceutical companies or other agencies for writing this article.

## Results

### Description of families

A total of 271 families in which the proband harboured a *BRCA1* and 301 families with *BRCA2* PV were eligible for inclusion in the analysis (eTable 2). Majority of the probands were of Chinese ancestry (52–81% depending on the study) except for the ovarian cancer case-series where the majority were Malays (eTable 3). There were 1121 and 1275 female first-degree family members in *BRCA1* and *BRCA2* families, respectively, of whom 144 and 65 family members in *BRCA1* families were diagnosed with breast and ovarian cancer, respectively, and the corresponding number in *BRCA2* families were 152 and 19 (eTable 4). The most common recurrent *PV* in *BRCA1* was c.2726dup (7% of *BRCA1* families) and in *BRCA2* was c.2808_2811del (5% of *BRCA2* families) (eTable 5, eFigure 1).

### Relative risk estimates

When the RR was assumed to be independent of age, the estimated *BRCA1* breast and ovarian cancer RRs (95% CI) were 15.6 (12.6–19.4) and 39.8 (29.6–53.3), respectively, and the estimated *BRCA2* breast and ovarian cancer RRs (95% CI) were 10.3 (8.3–12.7) and 6.8 (3.9–11.9), respectively (Table 1). When age-specific RRs were considered, the breast cancer RRs for *BRCA1* PV carriers were higher before age 40 (RR (95% CI) was 25.1 (9.1–69.3) for age group 20–29 and 27.0 (18.9–38.9) for age group 30–39) and then decreased with increasing age (RRs (95% CIs) were 16.1 (11.4–22.8), 12.1 (7.9–18.5), 6.4 (2.8–14.8) and 10.1 (3.4–30.0) for age groups 40–49, 50–59, 60–69 and 70–79, respectively, p-value for trend = 0.018). The RRs for ovarian cancer increased from 8.7 (0.9–79.4) in age group 20–29 to 81.7 (47.0–141.9) in age group 60–69, then decreased to 25.6 (5.5–119.5) in age group 70–79, but estimates were associated with wide confidence intervals (p-value for trend = 0.064). For *BRCA2*, the estimated breast cancer RR (95% CI) increased to a maximum of 14.1 (10.3–19.4) in the 40–49 age group and decreased to 4.9 (1.5–15.7) in the 70–79 age group (p-value for trend = 0.06). The ovarian cancer RRs increased from 2.3 (0.3–17.7) in age group 40–49 to 29.5 (10.1–86.2) in age group 70–79 (p-value for trend <0.001). The age-specific RR models provided a better fit to the data than the constant RR models (LRT: p-value = 7 × 10^−5^ for *BRCA1* and p-value = 0.002 for *BRCA2*).

**Table 1:** Estimated relative risk of breast and ovarian cancer for PV carriers of *BRCA1* or *BRCA2.*

Model	Breast cancer				Ovarian cancer			
	BRCA 1		BRCA 2		BRCA 1		BRCA 2	
	No. affected relatives	RR (95% CI)	No. affected relatives	RR (95% CI)	No. affected relatives	RR (95% CI)	No. affected relatives	RR (95% CI)
**Constant RR across age**								
20–79	144	15.6 (12.6–19.4)	152	10.3 (8.3–12.7)	64	39.8 (29.6–53.3)	19	6.8 (3.9–11.9)
**RR varied by age**								
20–29	6	25.1 (9.1–69.3)	3	9.7 (2–46.1)	1	8.7 (0.9–79.4)	0	–
30–39	38	27 (18.9–38.9)	21	9.5 (5.6–16.1)	5	10.3 (3–35.1)	0	–
40–49	47	16.1 (11.4–22.8)	60	14.1 (10.3–19.4)	16	35.5 (20.5–61.4)	3	2.3 (0.3–17.7)
50–59	38	12.1 (7.9–18.5)	47	10.9 (7.6–15.6)	23	51.9 (32.0–84.2)	3	6.3 (2.2–18.6)
60–69	10	6.4 (2.8–14.8)	16	4.8 (2.4–9.8)	16	81.7 (47.0–141.9)	9	15.8 (6.4–38.9)
70–79	5	10.1 (3.4–30.0)	5	4.9 (1.5–15.7)	3	25.6 (5.5–119.5)	4	29.5 (10.1–86.2)
**p-value**		**0.018**^a^		0.060^b^		0.064^b^		**0.0056**^a^

RR, Relative risk (with non-carriers as reference group); CI, Confidence Interval.

a Likelihood ratio test comparing logRR as a linear function of age against the model with a constant relative risk, df = 1.

b Likelihood ratio test comparing logRR as a piecewise function of age against the model with a constant relative risk, df = 2. See Supplementary methods.

### Analyses by country, ethnicity, PV location, ascertainment method

There was no evidence that breast and ovarian cancer RRs were different between countries, between ethnic groups or between ascertainment methods for PV carriers in either gene (eTable 6). Since the gender of unaffected children and the year of birth of unaffected siblings were not collected for Singapore breast cancer case-series families, we repeated the ethnic-specific analyses by using data from Malaysia only. There was no appreciable difference in results compared to the full cohort (eTable 7).

Compared to the region bounded by position c.2282 to c.4071, carriers with *BRCA1* PVs outside this region had breast cancer RRs between 1.4 and 1.5 and carriers with PVs in the upstream region (5′ to c.2830) had ovarian cancer RR of 1.3. Compared to carriers of *BRCA2* PVs in the OCCR (c.2831 to c.6401), carriers with PVs outside the OCCR had breast and ovarian cancer RRs of 0.4–0.8. However, none of these results were statistically significant (eTable 6).

### Age-specific incidences and cumulative risks

**Fig. 1:**
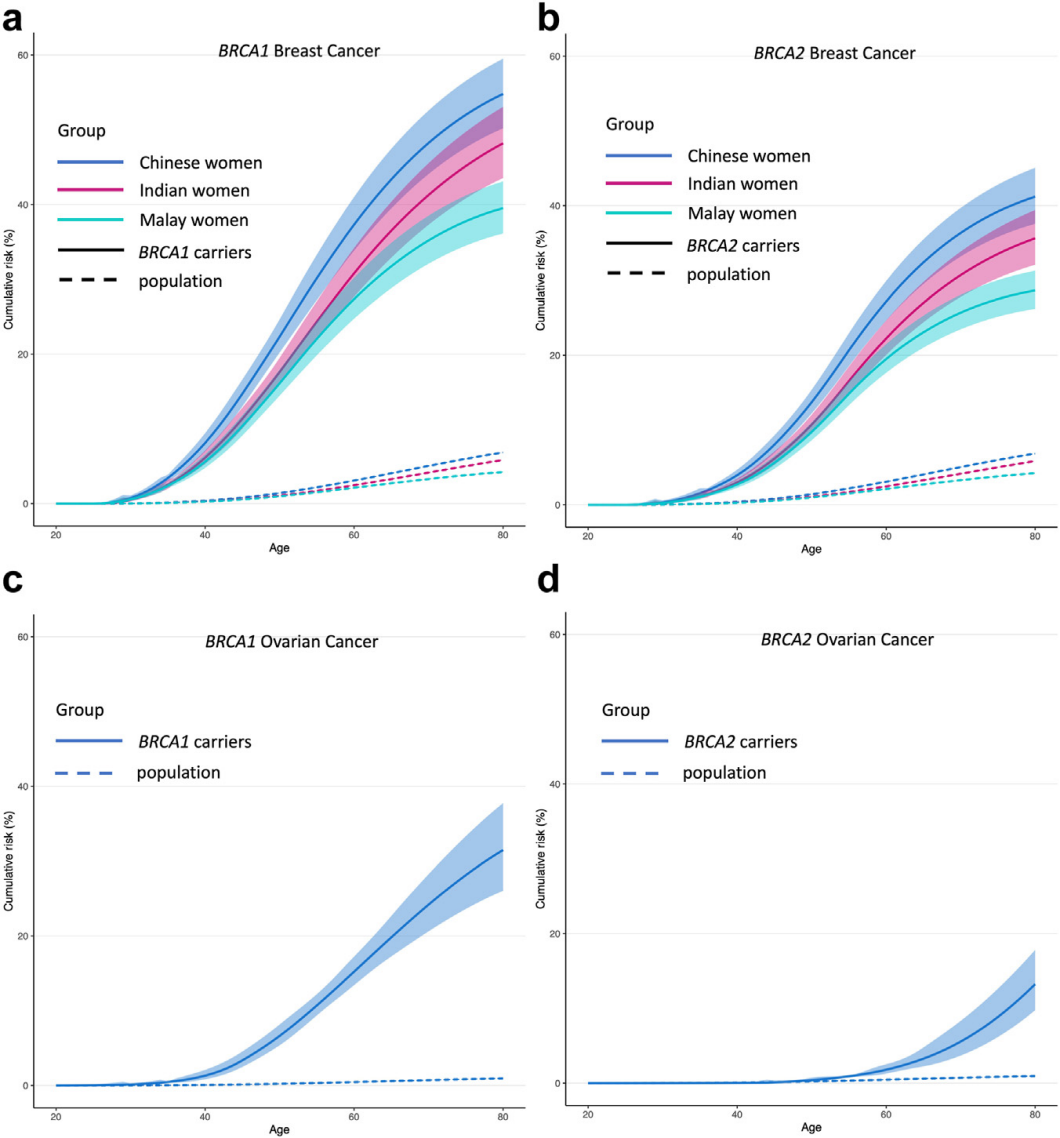
**Estimated cumulative risk of developing breast and ovarian cancer (under a model with no cohort effect) for women with germline *BRCA1* and *BRCA2* pathogenic variants**. Assume that population incidences are applicable to individuals born between 1950 and 1959 from Malaysia. Solid lines represent cumulative risk of *BRCA1* or *BRCA2* carriers; dashed lines represents cumulative risk of general population; Blue, red and green lines represent Chinese, Indian and Malay women, respectively. The shaded areas show the 95% CIs.

Using the ethnic-specific breast cancer incidences experienced by a woman born in Malaysia between 1950 and 1959, the breast cancer incidences for *BRCA1* carriers across all three ethnicities increased from age 20 to age 55, then remained similar between ages 55 and 65, and appeared to drop thereafter. A similar pattern was seen for *BRCA2* carriers (Table 2). The cumulative risk (95% CI) of breast cancer to age 80 was highest in Chinese-ancestry women followed by Indian-and Malay-ancestry women (Chinese versus Indian versus Malay: 55% (51–60%) versus 49% (44%–53%) versus 40% (36–44%) for *BRCA1* (pairwise p-values <0.05), and 42% (38–45%) versus 36% (33%–40%) versus 29% (26–32%) for *BRCA2* (pairwise p-values <0.05 except for Chinese versus Indians where p-value was 0.07) (Fig. 1a and b and Table 2).

**Table 2:** Breast and ovarian cancer incidences and cumulative risks of carriers of PVs in *BRCA1* and *BRCA2*.

Ages	Incidence per 1000 person-years (95% CI)				Cumulative risk, % (95% CI)			
	Breast^a^			Ovarian^b^	Breast^a^			Ovarian^b^
	Chinese	Malay	Indian		Chinese	Malay	Indian	
***BRCA 1* mutation carriers**								
20	0	0	0	0	0	0	0	0
25	0	0	0	0	0	0	0	0
30	3 (2–4)	2 (1–3)	2 (1–3)	0 (0–1)	1 (1–1)	1 (0–1)	1 (0–1)	0
35	7 (5–11)	5 (4–7)	6 (4–8)	1 (0–3)	3 (3–4)	2 (2–3)	3 (2–3)	0 (0–1)
40	12 (8–17)	8 (6–11)	9 (6–13)	2 (1–4)	8 (7–9)	6 (5–6)	6 (6–7)	1 (1–2)
45	17 (12–24)	12 (8–17)	13 (9–18)	6 (3–10)	15 (13–16)	10 (9–12)	11 (10–13)	3 (3–4)
50	20 (13–31)	14 (9–22)	16 (10–24)	8 (5–13)	23 (21–25)	16 (15–18)	18 (16–19)	7 (6–8)
55	22 (14–33)	15 (10–23)	18 (12–27)	10 (6–16)	30 (28–33)	22 (20–24)	25 (23–27)	11 (9–12)
60	22 (9–50)	14 (6–32)	18 (8–42)	11 (6–19)	38 (35–41)	28 (25–30)	31 (29–34)	15 (13–17)
65	20 (9–46)	12 (5–27)	17 (7–39)	11 (6–19)	44 (40–47)	32 (29–35)	37 (34–40)	20 (18–22)
70	17 (6–51)	10 (3–29)	15 (5–45)	11 (2–51)	49 (45–53)	35 (32–39)	42 (38–45)	24 (21–27)
75	14 (5–42)	7 (2–22)	13 (4–39)	10 (2–47)	52 (48–57)	38 (35–42)	46 (41–50)	28 (24–32)
80	11 (4–32)	5 (2–14)	11 (4–31)	8 (2–40)	55 (51–60)	40 (36–44)	49 (44–53)	31 (27–36)
***BRCA 2* mutation carriers**								
20	0	0	0	0	0	0	0	0
25	0	0	0	0	0	0	0	0
30	1 (1–2)	1 (1–1)	1 (1–2)	0	0 (0–1)	0 (0–0)	0 (0–0)	0
35	3 (2–6)	2 (1–4)	3 (2–4)	0	2 (1–2)	1 (1–1)	1 (1–1)	0
40	6 (5–9)	4 (3–6)	5 (4–7)	0	4 (3–5)	3 (2–3)	3 (3–4)	0
45	11 (8–14)	7 (5–10)	8 (6–11)	0 (0–3)	8 (7–9)	6 (5–6)	6 (6–7)	0
50	15 (10–21)	10 (7–15)	12 (8–17)	1 (0–2)	14 (13–15)	10 (9–11)	11 (10–12)	0 (0–1)
55	18 (13–27)	13 (9–18)	15 (10–22)	1 (0–4)	21 (19–23)	15 (14–16)	17 (15–18)	1 (1–1)
60	17 (8–34)	11 (5–21)	14 (7–28)	2 (1–5)	28 (25–30)	20 (18–22)	23 (21–25)	2 (1–2)
65	14 (7–28)	8 (4–17)	12 (6–24)	4 (1–9)	33 (30–36)	23 (21–26)	27 (25–30)	3 (3–4)
70	11 (3–35)	6 (2–20)	10 (3–31)	5 (2–14)	37 (34–40)	26 (24–28)	31 (28–34)	5 (4–7)
75	8 (3–26)	4 (1–13)	7 (2–24)	7 (3–20)	40 (36–43)	28 (25–31)	34 (31–37)	8 (7–11)
80	6 (2–18)	3 (1–8)	6 (2–18)	10 (4–26)	42 (38–45)	29 (26–32)	36 (33–40)	12 (10–15)

a Incidences and cumulative risks for breast cancer were calculated based on population calendar, cohort and ethnic-specific breast cancer incidences for a woman born in Malaysia between 1950 and 1959. Disease-specific mortality was not available and hence was not accounted for in cumulative risk estimates.

b Incidences and cumulative risks for ovarian cancer were calculated based on overall population calendar and cohort-specific ovarian cancer incidences for a woman born in Malaysia between 1950 and 1959, as ethnic-specific ovarian cancer incidence was not available. Disease-specific mortality was not available and hence was not accounted for in cumulative risk estimat.

**Fig. 2:**
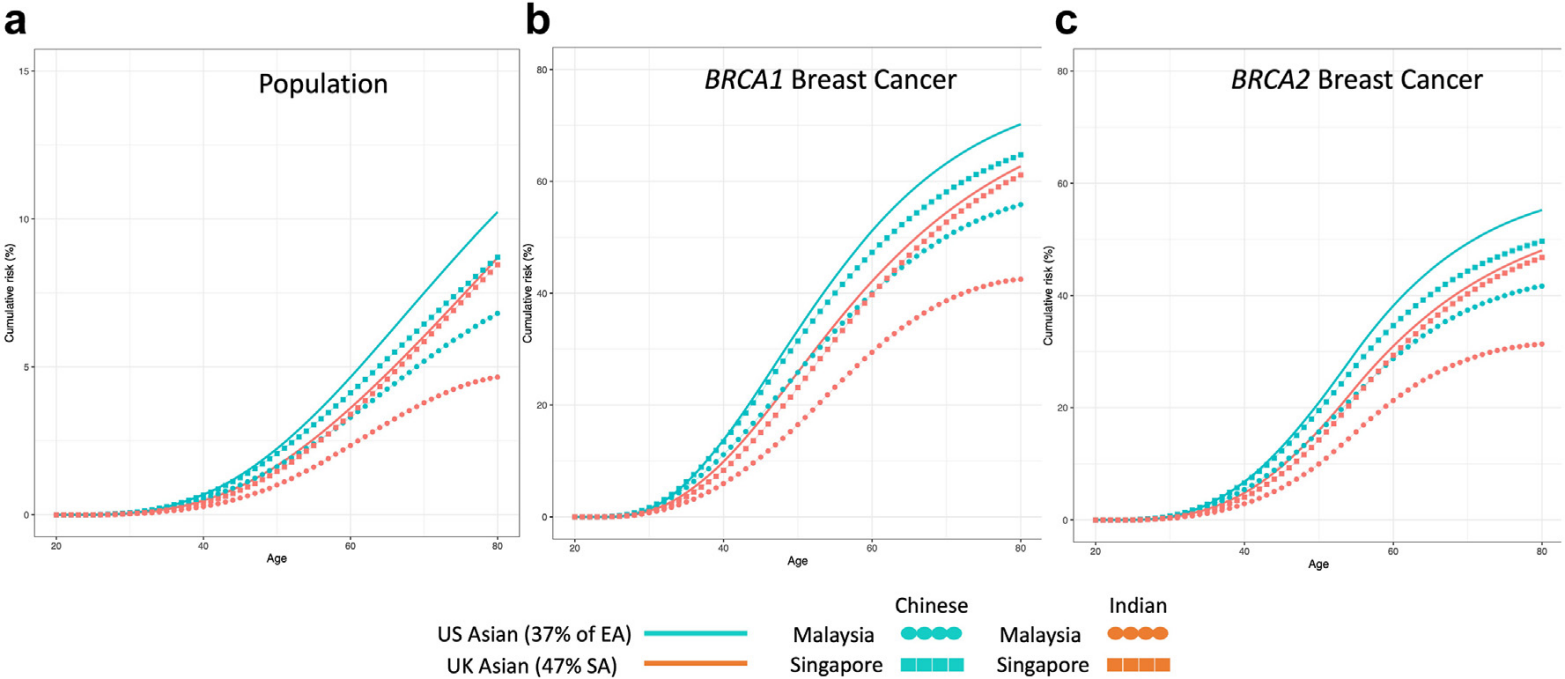
**Estimated cumulative risk of breast cancer (under a model with no cohort effect) for Chinese and Indians in Malaysia and Singapore, and Asians in UK and USA**. Assume that estimated relative risks are applicable to Asian populations in the UK and US, and women had the incidences as reported between 2016 and 2018 for UK, 2015–2019 for US, and 2014–2016 for Malaysia and Singapore. Blue line, blue circle and blue square represent the cumulative risk of US Asians, Malaysian Chinese and Singaporean Chinese, respectively while orange line, orange circle and orange square represent UK Asians, Malaysian Indians and Singaporean Indians, respectively.

Assuming that age-specific RRs are constant across Asian populations in any country and using the latest available incidences for women of Chinese and Indian-ancestry from Malaysia and Singapore as well as Asians from UK and USA, Fig. 2 aims to demonstrate how cumulative breast cancer risks can vary within the same ethnic groups due to differences in population cancer incidences across different countries. The ethnic-specific population cancer incidences for Asian populations in the USA and UK were not available, thus we made assumption based on the demographic composition of these countries. The cumulative risks of Chinese in Singapore were similar to Asians in USA (with approximately 37% East Asians in the USA^25^) but higher compared to Chinese in Malaysia. The cumulative risks of Indians in Singapore were similar to Asians in UK (with approximately 47% South Asians in the UK^26^) but higher compared to Indians from Malaysia.

Using the ovarian cancer incidences experienced by a woman born in Malaysia between 1950 and 1959, the ovarian cancer incidences for *BRCA1* carriers increased with age up to 60 years old and remained similar between ages 60 and 80. The ovarian cancer incidences for *BRCA2* carriers were lower compared to *BRCA1* carriers (incidences per 1000 person-years increased from 2 at age 60 to 11 at age 80) (Table 2). The cumulative risk (95% CI) of ovarian cancer to age 80 was 31% (27–36%) for *BRCA1* carriers and 12% (10–15%) for *BRCA2* carriers (Table 2 and Fig. 1c and d). Ethnic-specific population ovarian cancer incidences were not available to determine ethnicity-specific cumulative risk estimates.

### Birth cohort effect

**Fig. 3:**
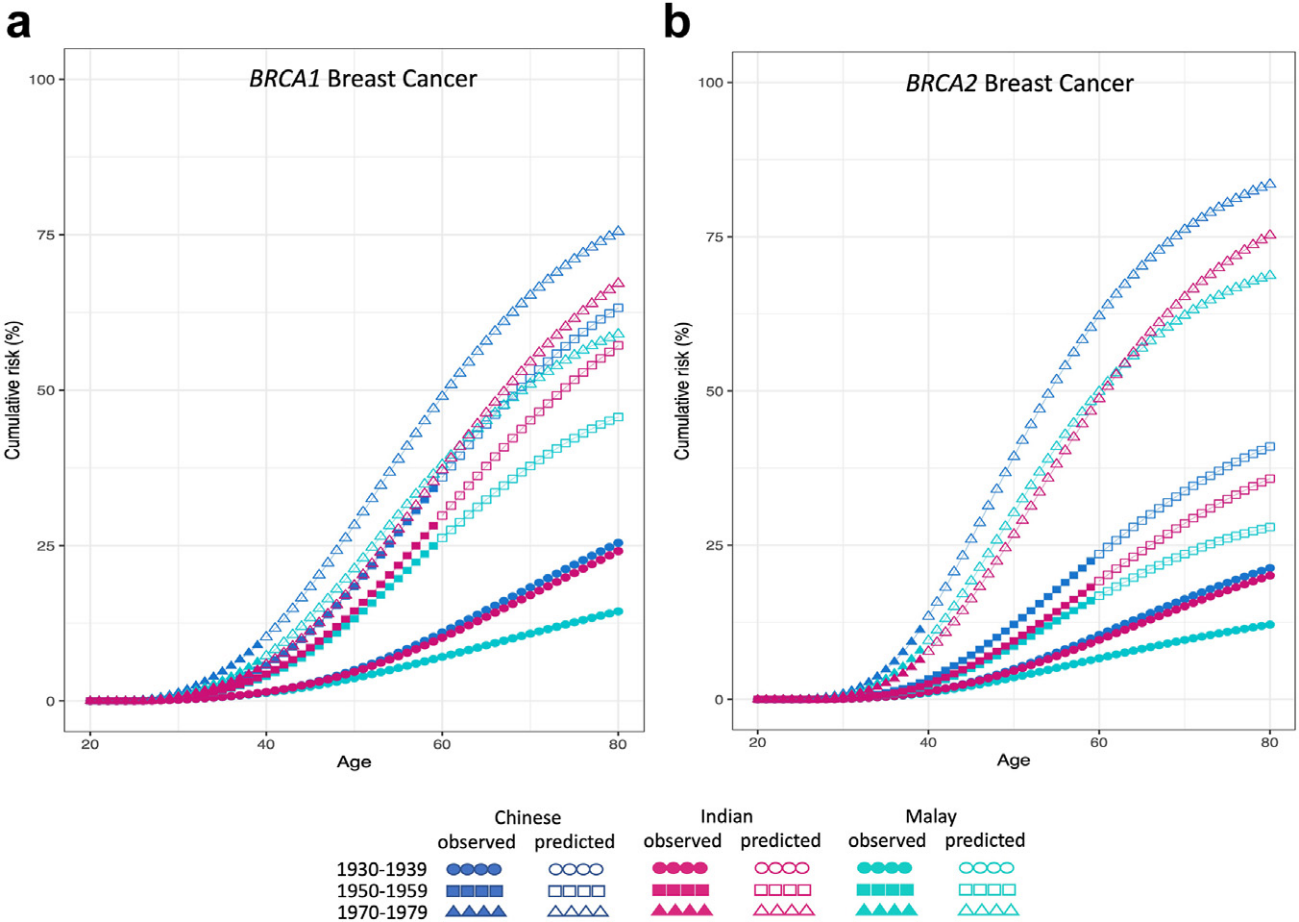
**Estimated cumulative risk of developing breast cancer (under a model that allows for cohort-specific relative risks) for women with germline(a)*BRCA1* and(b)*BRCA2* pathogenic variants**. Circle, square and triangle represent birth cohort 1930-1939, 1950-1959, and 1970-1979, respectively, where solid symbol represents observed risk and hollow symbol represents predicted risk; blue, red and green represent Chinese, Indian and Malay ethnicity, respectively.

We investigated whether breast and ovarian cancer risks varied by birth cohort: before 1940, 1940–59, 1960–69, and after 1969. For *BRCA1*, relative to the before-1940 cohort, the breast cancer RRs (95% floating-CI^27^) were 2.5 (1.8–3.4), 2.9 (1.9–4.6) and 3.3 (1.9–5.7) for the three later birth cohorts, respectively (eTable 6). The corresponding estimates for ovarian cancer were 7.8 (5.3–11.6), 10.7 (5.2–22.1), and 5.7 (1.1–30.5). For *BRCA2*, the corresponding cohort effect for breast cancer were of 1.4 (1.1–2.2), 2.2 (1.4–3.4) and 4.4 (2.3–8.5). The ovarian cancer RR (95% floating-CI) was 3.3 (1.2–10.2) for *BRCA2* carriers born in 1940–1959 compared to those who were born before 1940. The cohort-specific model provided a significantly better fit than the age-only model (LRT: p-value = 3.6 × 10^−5^ for *BRCA1* and 0.018 for *BRCA*2). Under the birth cohort-specific models, the cumulative risk of breast cancer to age 80 was predicted to be higher in PV carriers born in 1970–79 compared to PV carriers born in 1930–39, across all three ethnicities (Fig. 3a and b).

## Discussion

In this study, we have defined breast and ovarian cancer risk estimates for *BRCA1* and *BRCA2* PV carriers in Malaysia and Singapore. These estimates refine estimates for carriers of Chinese ancestry and quantify for the first time the risks for carriers of Malay and Indian ancestry. We found that the age-specific RRs for *BRCA1* and *BRCA2* PV carriers were similar across ethnic groups.

Our study showed that breast cancer RRs vary substantially by age, from approximately 25 for *BRCA1* and 10 for *BRCA2* at age <30 to 10 for *BRCA1* and 5 for *BRCA2* at age >70. The patterns are largely similar to those reported in women of European ancestry,^1^ except for age group 40–49 in *BRCA1* PV carriers where the breast cancer RRs appeared to be lower compared to European studies (eTable 8). The estimated breast cancer incidences for all ethnicities peak at age 55 then decrease again thereafter for both genes (Table 2 and eTable 9). This pattern is somewhat different from that reported in European populations, where peak incidence occurs earlier (at 45 for *BRCA1* carriers and 50 for *BRCA2* carriers), plateauing thereafter.^1^^,^^6^ Comparing to the higher breast cancer RRs at younger age for both *BRCA1* and *BRCA2* carriers, the RRs for ovarian cancer increased with age for both mutations. This pattern is different from that reported in European populations, where RR peaked between age 40–49 for *BRCA1* carriers and 50–59 for *BRCA2* carriers, though the confidence intervals between the two studies overlapped (eTable8).

The cumulative breast cancer risks of Asian carriers vary depending on underlying population-specific cancer incidences. The corresponding risks to age 70 are substantially lower for Asian carriers in Malaysia than those reported for European women: 40–55% (depending on ethnicity) versus 65% for *BRCA1* carriers and 29–42% (depending on ethnicity) versus 45% for *BRCA2* carriers).^1^ These differences in absolute risks reflect the lower population incidences in Malaysia versus Western countries and are consistent with the hypothesis that the risks conferred by PVs combine multiplicatively with the effects of other risk factors (such as lifestyle or genetics factors), and are largely independent of genetic ancestry.^13^ A similar observation was made for women of African ancestry based on a study from Ghana.^28^ It is also worth noting that the predicted absolute risks in Singapore, where population incidences are closer to those of European ancestry, are notably higher than those based on Malaysia (57–61% for *BRCA1* carriers and 43–47% (depending on ethnicity) for *BRCA2* carriers; Fig. 2 and eTable 9). Assuming that RRs are constant across Asian populations in any countries, the absolute risks of Asian carriers in Western countries is close to that of European carriers, whereas the absolute risks of Asian carriers in Asian countries with lower population risk will be lower than that reported here for Malaysians (Fig. 2).

Notably, we found a significant birth cohort effect, likely driven by the substantial increase in breast cancer incidence largely driven by urbanisation and significant changes in reproductive patterns in Asia.^29^, ^30^, ^31^ Even after adjusting for birth-cohort-specific population cancer incidences, breast cancer risk among carriers of PVs has increased over time (Fig. 3 & eTable 6). Although lower life expectancy and under-reporting of cancer cases in earlier decades, and higher uptake of screening for recent generations can at least partly explain this observation,^32^ the data could also point to the influence of lifestyle, reproductive and hormone-related modifiers on disease risks for PV carriers.^33^, ^34^, ^35^ In this regard, risk assessment tools which integrate genetic modifiers with lifestyle, hormonal or other cancer risk factors could be important for refining personalised breast cancer risks in Asian carriers.

For ovarian cancer, the cumulative risk in carriers of *BRCA1* PV is low under the age of 45 years but reaches more than 10% by the age of 55 years, whereas it remains low until the age of 65 years for *BRCA2* mutation carriers. The recommended ovarian cancer control guidelines include discussion of risk-reducing salpingo-oophorectomy (RRSO) at ages 35–40 for *BRCA1* carriers and 40–45 years for *BRCA2* carriers.^36^ However, given the lower risk in *BRCA2* carriers in Asian countries, it may be appropriate to delay this surgical prevention to avoid the potential consequences of premature surgical menopause.^37^^,^^38^ Nevertheless, the confidence intervals for the ovarian cancer RR estimates for *BRCA2* carriers are wide, larger studies are needed to improve the precision of these estimates.

Our study has several limitations. While we confirmed cancer family history on all family members who underwent genetic testing, we relied on proband-reported family history on other members and hence may be susceptible to inaccuracies. Although studies have shown high accuracy of proband-reported family history of breast cancer, accuracy has been shown to be lower for less common cancers, including ovarian cancer.^39^^,^^40^ A second limitation would be high proportion (∼33%) of missing information on year of birth, mostly from the Singapore breast cancer case-series where the information on year of birth was not collected for unaffected siblings of proband. An additional limitation is an assumption that unaffected children recruited in the Singapore breast cancer case-series were all females. To access the impact of these limitations, we performed sensitivity analyses to examine how RRs vary across birth cohorts and ethnicities without using the Singapore data and the results remained similar. Another limitation is ethnic-specific ovarian cancer incidence was not available for adjustment. However, additional ethnic-specific parameters included in the models were not significant, suggesting that not adjusting for ethnic-specific population ovarian cancer incidences is unlikely to have resulted in biased RR estimates. It is also important to acknowledge that the RR estimates for the birth cohort >1970 have relatively wide confidence intervals, indicating greater uncertainty in the estimated cumulative risks for the birth cohort 1970–79 based on the birth-cohort model alone. Finally, although information on salpingo-oophorectomy and bilateral mastectomy for relatives were not available, the phenotypic data on relatives were collected at or prior to genetic testing. Hence it is unlikely that these surgeries would have taken place prior to genetic testing results in family members. Moreover, the rate of both of these surgeries have been historically low in Asia and therefore unlikely to impact on the parameter estimates.

In summary, we have shown that the age-specific RR estimates of *BRCA1* and *BRCA2* carriers of Asian ancestry were similar to those previously observed in European populations, but the corresponding absolute breast and ovarian cancer risks vary depending on the underlying population- and birth-cohort specific cancer incidences. The current European-derived risk management strategies may be appropriate for Asian carriers in developed countries where absolute risks are expected to be similar to Europeans. By contrast, the current European-derived risk management strategies may require refinement in Asian populations with lower risk of these cancers as the current absolute risk estimates are significantly higher than appropriate. It may be critical to develop risk assessment tools which integrate genetics, lifestyle, hormonal and other risk factors for refining population- and age-specific cancer risks, particularly for these Asian carriers in developing countries.

## Contributors

Conceptualization: W.K.H., D.F.E., A.C.A., S.H.T.; Resources: J.C., Z.Z., SGBCC investigators, M.H.S., M.K.T., Y.L.W., MaGiC Investigators, A.M.D., M.H., C.H.Y., N.A.M.T., J.Li., J.N., S.H.T.; Data curation: N.T.H., S.Y.Y., J.Lim, N.D.B.I., P.J.H., E.A.W., P.P.S.N., C.L., J.A., M.C.T.; Formal analysis: W.K.H., X.Y., D.F.E., A.C.A., S.H.T.; Validation: W.K.H., A.C.A., S.H.T.; Manuscript writing—original draft: W.K.H., D.F.E., J.N., A.C.A., S.H.T.; Manuscript writing—review and editing: all authors.

## Data sharing statement

Request for access to data from the Malaysian studies, Singapore SGBCC study and Singapore NCC study can be made via submission of an inquiry to Prof. Soo-Hwang Teo at genetics@cancerresearch.my, Dr. Mikael Hartman at mikael_hartman@nuhs.edu.sg, and Dr. Joanne Yuen-Yie Ngeow at joanne.ngeow@ntu.edu.sg, respectively.

## Declaration of interests

Z.Z received honorarium from AstraZeneca. J.N received research funding from AstraZeneca and MiRXES. A.C.A is listed as creator of the BOADICEA model which has been licensed by Cambridge Enterprise, from which University of Cambridge may receive royalties. N.A.M.T received honoraria for lectures from Zuellig Pharma Sdn Bhd and Astra Zeneca, received support for attending meetings and/or travel from MSD and Astra Zeneca. S.Y.Y received speaker's honoraria from Astra Zeneca, she is the president of Genetic Counselling Society Malaysia.

## Appendix A Supplementary data

Supplementary files Tables S5

Supplementary information_revision

Supplementary material_Names of Investigators within MaGiC and SGBCC
