# Supplementary material for: Age-specific breast and ovarian cancer risks associated with germline BRCA1 or BRCA2 pathogenic variants – an Asian study of 572 families
Source: Lancet Reg Health West Pac. 2024 Feb 5;44:101017. doi: 10.1016/j.lanwpc.2024.101017 (PMC10851205; doi:10.1016/j.lanwpc.2024.101017)
Supplement: Supplementary information_revision [file mmc2.docx]

**Supplementary Material**

Supplementary Methods……………………………………………………………………….2

eTable 1: Previously published breast cancer ovarian cancer risk associated with PV in *BRCA1* and *BRCA2*………………………………..…………………………………………..5

eTable 2: List of participating studies by ascertainment scheme and gene mutated……….....6

eTable 3: Characteristic of family by ascertainment method and gene mutated……………...7

eTable 4: Summary of disease status (first cancer) of family member by gene mutated of proband, recruitment country and age of diagnosis (or age of censoring)……………...…….8

eTable 6: Effect of birth cohort and ethnicity on breast and ovarian cancer risk of carriers of PV in *BRCA1* and *BRCA2* from Malaysia and Singapore…………………………………...9

eTable 7: Effect of birth cohort and ethnicity on breast and ovarian cancer risk of carriers of PV in *BRCA1* and *BRCA2* from Malaysia…………………………………………………...10

eTable 8: Estimated relative risk of breast and ovarian cancer for PV carriers of *BRCA1* or *BRCA2*………………………………………………………………………………………..11eTable 9: Breast and ovarian cancer incidences and cumulative risks of carriers of mutations in BRCA1 and BRCA 2 (Singapore)………….……………………………………………..13

eFigure 1: Lollipop plot of probands with (A) *BRCA1* and (B) *BRCA2* mutations….……..14

eFigure 2: Principal components plot of index cases ………………………………...……..15

Supplementary files description……………………………………………………………...16

Consortia memberships………………………………………………………………………16

References……………………………………………………………………………………17

**Supplementary Methods**

**Families**

Data on families with *BRCA1* and *BRCA2* PV from Malaysia and Singapore were obtained through two ascertainment methods: (1) Four case-series unselected for cancer family history- (a) one breast cancer hospital-based study in Malaysia (MyBrCa)^1^ and one in Singapore (SGBCC)^2^; one hospital- (MyOvCa)^3^ and one population-based (MaGic)^4^ study of ovarian cancer cases in Malaysia;, (2) breast and ovarian cancer patients from multiple-case families recruited through genetics clinics in Malaysia (MyF) and Singapore (NCCS). Patients in NCC study were referred to genetic testing if they have metastatic cancer (hereafter denoted as NCC1) or have multiple cancer cases in the families (hereafter denoted as NCC2). The breast and ovarian cancer patients recruited through the case-series and the first affected family member recruited through multiple-case families was designated as the proband. All participants provided informed consent for genetic testing, provided a blood or saliva sample and were asked to complete a structured questionnaire which included detailed information on all first and second degree relatives, except SGBCC where only family history of first-degree relatives was available. Genetic counselling and predictive genetic testing was offered to other family members once a pathogenic variant was identified in the proband: all relatives who were tested provided informed consent. For SGBCC, carriers were referred for genetic counselling but follow-up information was not available as genetic counselling was not always available in the same hospital in which the case was recruited into the study. Families with probands who were not of self-reported Chinese, Malay or Indian ancestry and families with no additional information beyond the information relevant to ascertainment were excluded from this study. The number of eligible families by gene and recruitment scheme are detailed in eTable 2. eFigure 2 shows the principal components plot stratified by self-reported ethnicity using index cases from MyBrCa, SGBCC, MyOvCa, MaGic and MyF studies. For these studies, genotyping data using OncoArray are available and principal components were generated as part of OncoArray Consortium as previously described^5^.

Library preparation and sequencing of MyBrCa and SGBCC have been previously described ^6,7^. In brief, germline DNA were sequenced using targeted sequencing panels that target the coding regions and exon-intron boundaries of known and candidate breast cancer risk genes including *BRCA1* and *BRCA2*. Library preparation was conducted using one of the two systems – Fluidgm Access Array or Juno system, and were sequenced on Illumina HiSeq 2500 or HiSeq 4000. Variant calling was done by VarDict ^8^ and Genome Analysis Toolkit (GATK; https://www. broadinstitute. org/gatk) and were considered pathogenic or likely pathogenic (collectively referred hereafter as pathogenic) if they resulted in a truncated protein or have been previously reported as disease-associated (by ClinVar (https://www.ncbi.nlm.nih.gov/clinvar/) or ENIGMA BRCA1/2 expert panel guidelines (<https://enigmaconsortium.org/)>) based on ACMG/AMP Guidelines. Library preparation and sequencing MyOvCa, MaGiC and MyF studies were performed using an amplicon‑based Hi-Plex-NGS method on an Illumina MiSeq, as previously described ^3,9^. NCCS patients are referred for HBOC testing and undergo CLIA and CAP certified commercial clinical multi-gene panel testing. Confirmatory Sanger Sequencing was carried out on all pathogenic variants.

**Statistical Methods**

*Missing information*

The following information was collected on family members: exact family relationships, gender, vital status, year of birth , age and year of death, age at last follow up (for those who do not underwent genetic testing, age of last follow up is equivalent to year of proband was last followed up minus year of birth), age at breast, ovarian, other cancer diagnosis, *BRCA1* and *BRCA2* pathogenic variant status (carrier, non-carrier, not tested). For families recruited through SGBCC, gender information for unaffected children and information on year of birth for unaffected siblings were not available as these information were not collected. All unaffected children from SGBCC were assumed to be female. Family members were assumed to have the same ethnicity as probands. Missing year of birth was inferred using (a) year of birth of parents/children plus/minus average age at pregnancy estimated from parous women without missing year of birth, (b) or mean year of birth of family members of the same generation, (c) or the mean age of family members of nearest generation assuming the average generation gap was 20 years. Missing age of death was replaced by average age of death of family members of the same generation or the age at last follow-up if available. For individuals with a missing age of diagnosis, this was replaced by the age at last follow up, age of death or age 80 whichever occurred first.

*Censoring*

Family members were censored at the age at first breast or ovarian cancer diagnosis, age at death, age at last follow-up or age 80 years, whichever occurred first. Individuals with no age information were censored at age 0 and they did not contribute to the analysis.

*Ascertainment adjustment*

Data for each family were divided into two parts – part 1 (D1) comprised of data relevant to ascertainment and part 2 (D2) comprised of data not relevant to ascertainment. For population/hospital-based case-series unselected for family history, D1 consisted of phenotype and genotype information of probands while D2 consisted of the phenotypic and genotypic information of family members of probands. For families ascertained through multiple affected family members, D1 consisted of genotypic information of probands and phenotypic information of all family members that led to the ascertainment while D2 consisted of genotypic information of all family members (eTable 2). For parameters estimation, we maximised the likelihood by conditioning on information in D1, i.e. the conditional likelihood is given by P(D1,D2|D1), where P(D1,D2|D1) is the probability of observing data in the entire pedigree given the data that led to ascertainment. This method has been previously described ^10-12^.

*Genetic models*

For the main analysis, the cancer incidence for individual *i* at age *t* was assumed to be country-, ethnic- and birth cohort-specific, and was assumed to follow a model of the form:

$\lambda_{i}\left( t,c,w \right)=\lambda_{0}\left( t,c,w \right) exp(\left( t \right)G_{i}$)

where $\lambda_{0}\left( t,c,w \right)$ is the baseline cancer incidence at age *t* for non-carriers in birth cohort *c* and ethnic group *w*, $\left( t \right)$ is the age-specific log RR in carriers compared to non-carriers, $G_{i}$ is 1 for carriers and 0 for non-carriers. Conditional on the genotype, the probability of developing breast cancer was assumed to be independent of the probability of developing ovarian cancer. The baseline cancer incidence was estimated by constraining the overall cancer incidence over carriers and non-carriers in the model to agree with ethnic- and birth cohort-specific population age-specific incidences ^13^. The ethnicity-specific incidences reported in the country where the participants were recruited were used.

We considered models in which: (1) the log RR was assumed to be constant, that is $\beta\left( t \right)=\beta$; and (2) the log RRs were assumed to be constant within 10-year age-interval (20-29, 30-39, 40-49, 50-59, 60-69, 70-79). To check the evidence of trend in age-specific RRs, we modelled the log RRs as a function of age using linear and piecewise models, model with lower AIC will be selected and compared against model with assumed constant RRs. Z-test was used to compare age-specific log RRs estimated from this study with those previously reported in European studies. We also considered country-, birth cohort- and ethnicity-specific models, where an additional variable for each subgroup (assumed to be constant over all ages) was included in the model to allow for log RRs to differ between subgroups. Floating confidence intervals were reported for the subgroup analyses with more than two categories ^14^. A similar method was used to explore variation in cancer risk by ascertainment methods and by the location of the PV within the *BRCA1* and *BRCA2* genes. Mutations in *BRCA1* were grouped into 3 regions (5′ to c.2281, c.2282 to c.4071, c.4072 to 3′) and mutations in *BRCA2* were grouped according to the definitions of the ovarian cancer cluster region 5′ to c.2830, c.2831 to c.6401, c.6402 to 3′) ^15^. Nested models were compared against each other using likelihood ratio test. The allele frequency for *BRCA1* and *BRCA2* was assumed to be 0.0007 and 0.001, respectively, estimated from the healthy women serving as controls for the hospital-based breast cancer case-series from Malaysia and Singapore ^6,7^.

*Cancer incidences*

Calendar-specific population incidences reported in five-year age intervals were available between 1968 to 2017 for Singapore ^16^ and between 1994 to 2016 for Malaysia ^17-20^. Since Singapore was one of the states in Malaysia before 1965, we assumed the cancer incidence in Malaysia between 1968 to 1977 to be the same as that reported in Singapore. The calendar-specific incidences for Malaysia between 1978-1993 were estimated using local weighted regression approach ^12^. Eight birth cohort-specific incidences were derived (<1920, 1920-1929, 1930-1939, 1940-1949, 1950-1959, 1960-1969, 1970-1979, >1979) and smoothed using local weighted regression approach before incorporated into the genetic models.

Calendar- and age-specific breast cancer incidences for UK Asians population were estimated by $\lambda_{0}\left( t \right) exp(\gamma\left( t \right)$), where $\lambda_{0}\left( t \right)$ is the age-specific population incidences for UK White population reported between 2016-2018 and $exp(\gamma\left( t \right)$) is the assumed to be 0.72 for age 0-64 and 0.71 for age 65-90 as estimated by *Delon et. al^21^.*

*Cumulative risk*

Cumulative risk of disease was calculated from the estimated cumulative incidence,

$$\Lambda_{i}\left( t,c,w \right)=\sum_{k=1}^{t} \lambda_{i}\left( k,c,w \right)$$

and the variance of $\Lambda_{i}\left( t,c,w \right)$, derived using the delta method, is given by

${\sum_{\left\{ k=1 \right\}}^{t} {(\lambda}_{0}\left( k \right)e^{\beta_{k}})}^{2}\mathrm{Var}\left( \beta_{k} \right)+2\sum_{\left\{ k>j \right\}} \lambda_{0}\left( k \right)e^{\beta_{k}}\lambda_{0}\left( j \right)e^{\beta_{j}}Cov(\beta_{k},\beta_{j})$.

The $\log\Lambda_{i}\left( t,c,w \right)$ was assumed to be normally distributed. The cumulative risk was therefore given by $1-\exp[-\Lambda_{i}\left( t,c,w \right)]$ and the corresponding 95% confidence interval (CI) was $1-\exp[-\exp\left( \log\Lambda_{i}\left( t,c,w \right)\pm1.96\sqrt{var\left( \log\Lambda_{i}\left( t,c,w \right) \right)} \right)]$. Z-test was used for pairwise comparison of cumulative risks between ethnicities or populations. To estimate the cumulative risk by birth cohorts, the variance of cumulative incidence is given by

${\sum_{\left\{ k=1 \right\}}^{t} {\{(\lambda}_{0}\left( k \right)e^{\beta_{k}})}^{2}\mathrm{Var}\left( \beta_{k} \right)+V\left( \gamma_{c} \right)(\sum_{\left\{ z=1 \right\}}^{i} \lambda_{0}\left( z \right)e^{\beta_{z}})^2+2\sum_{\left\{ k>j \right\}} \lambda_{0}\left( k \right)e^{\beta_{k}}$ $\lambda_{0}\left( j \right)e^{\beta_{j}}Cov\left( \beta_{k},\beta_{j} \right)+$ $\lambda_{0}\left( j \right)e^{\beta_{j}}Cov(\beta_{j},\gamma_{c})\sum_{\left\{ z=1 \right\}}^{i} \lambda_{0}\left( z \right)e^{\beta_{z}}$

where$\gamma_{c}$ is the log relative risk associated with the birth cohort of interest.

**Supplementary Tables**

**eTable 1: Previously published breast cancer and ovarian cancer risk associated with PV in *BRCA1* and *BRCA2*.**

| **Ascertainment Scheme** | **Country** | **Number of families** | | **Breast Cancer** | | | | **Ovarian Cancer** | | | |
| --- | --- | --- | --- | --- | --- | --- | --- | --- | --- | --- | --- |
|  |  | **BRCA1** | **BRCA2** | **BRCA1** | | **BRCA2** | | **BRCA1** | | **BRCA2** | |
|  |  |  |  | **Relative risk (95% CI)** | **Cumulative risk by age 70 (95% CI)** | **Relative risk (95% CI)** | **Cumulative risk**  **by age 70 (95% CI)** | **Relative risk (95% CI)** | **Cumulative risk**  **by age 70 (95% CI)** | **Relative risk (95% CI)** | **Cumulative risk**  **by age 70 (95% CI)** |
| Multiple-case families | Korea^22^ | 151 | 225 | 18.0  (3.0-103) | 49%  (11-98) | 11.0  (5.0-27.0) | 35%  (16-65) | - | - | - | - |
| Hospital-based case-series unselected for family history | China^23^ | 70 | 55 | 3.8  (2.3 - 6.1) | 40%  (24-54) | 4.4  (2.7 - 7.3) | 37%  (27-52) | - | - | - | - |
| Population-based | Hong Kong, China^24^ | 66 | 84 | 3.3  (2.5-4.4) | 54%  (35-72) | 3.3  (2.5-4.4) | 48%  (32-69) | NR | 22%  (10-27) | NR | 7.3%  (NR) |
| Hospital-based case-series unselected for family history | Japan^25^ | 127 | 254 | 16.1 (7.1-36.7) | 72%*  (20-91) | 10.9 (7.0-17.1) | 58%*  (38-72) | 75.6 (31.6-180.6) | 66%*  (13-86) | 11.3 (5.6-23.0) | 15%*  (5-24) |

NR: Not Reported

* cumulative risk by age 85.

**eTable 2: List of participating studies by ascertainment scheme and gene mutated.**

| **Ascertainment method** | **Proband** | **Studies** | **Country** | **Number of families recruited** | | **Number of families excluded^a^** | | **Number of families remained in the study** | | **Ascertainment adjustment** | |
| --- | --- | --- | --- | --- | --- | --- | --- | --- | --- | --- | --- |
|  |  |  |  | **BRCA1 (N = 352)** | **BRCA2 (N = 378)** | **BRCA1 (N = 80)** | **BRCA2 (N = 77)** | **BRCA1 (N = 271)** | **BRCA2 (N = 301)** | **D1: data relevant to ascertainment** | **D2: data not relevant to ascertainment** |
| Hospital-based case-series unselected for family history | Breast | MyBrCa | Malaysia | 85 | 118 | 0 | 1 | 85 | 117 | (1) Phenotype of proband  (2) Variant status of proband | (1) Phenotypes of all family members except proband  (2) Variant status of all family members except proband’s |
|  |  | SGBCC | Singapore | 38 | 75 | 0 | 1 | 38 | 74 |  |  |
|  | Ovarian | MaGiC | Malaysia | 43 | 24 | 2 | 0 | 41 | 24 |  |  |
|  |  | MyOvCa | Malaysia | 23 | 12 | 0 | 0 | 23 | 12 |  |  |
|  | Metastatic cancer | NCCS1 | Singapore | 53 | 51 | 5 | 9 | 48 | 42 |  |  |
| Multiple-case families | Breast/Ovarian | MyF | Malaysia | 13 | 10 | 6 | 5 | 7 | 5 | (1) Phenotypes of all family members  (2) Variant status of proband | (1) variant status of family members except proband’s |
|  |  | NCCS2 | Singapore | 97 | 88 | 68 | 61 | 29 | 27 |  |  |

^a^Families with proband who were not of self-reported Chinese, Malay or Indian, and families with no additional information beyond the information relevant to ascertainment.

**eTable 3: Characteristic of family by ascertainment method and gene mutated**

|  | **BRCA1** | **BRCA2** |
| --- | --- | --- |
|  | **(271 families)** | **(301 families)** |
| **Breast cancer case series unselected for family history** | | |
| Total families | 123 (45.4%) | 191 (63.5%) |
| Mean (SD) age of diagnosis of index cases | 42.2 (11.4) | 46.7 (11.3) |
| Ethnicity of index cases |  |  |
| Chinese | 64 (52.0%) | 126 (66.0%) |
| Malay | 34 (27.6%) | 40 (20.9%) |
| Indian | 25 (20.3%) | 25 (13.1%) |
| median no. (IQR) family members | 9 (IQR: 7-11) | 9 (IQR: 6-11) |
| No. of pedigree with one or more family members affected with breast/ovarian cancer | 64 (52.0%) | 69 (36.1%) |
| No. of pedigree with additional members tested for mutation | 26 (21.1%) | 27 (14.1%) |
| **Ovarian cancer case series unselected for family history** | | |
| Total families | 64 (23.6%) | 36 (12.0%) |
| Mean (SD) age of diagnosis of index cases | 48.8 (12.0) | 51.1 (15.0) |
| Ethnicity of index cases |  |  |
| Chinese | 22 (34.4%) | 10 (27.8%) |
| Malay | 32 (50%) | 19 (52.8%) |
| Indian | 10 (15.6%) | 7 (19.4)% |
| median no. (IQR) family members | 10 (IQR: 7-13) | 11 (IQR: 8-14) |
| No. of pedigrees with one or more family members affected with breast/ovarian cancer | 32 (50%) | 11 (30.6%) |
| No. of pedigrees with additional members (other than proband) tested for mutation | 13 (20.3%) | 6 (16.7%) |
| **Metastatic cancer case series unselected for family history** | | |
| Total families | 48 (17.7%) | 42 (14.0%) |
| Mean (SD) age of diagnosis of index cases | 50 (11.1) | 47.6 (10.3) |
| Ethnicity of index cases |  |  |
| Chinese | 32 (66.7%) | 28 (66.7%) |
| Malay | 11 (22.9%) | 8 (19.0%) |
| Indian | 5 (10.4%) | 5 (14.3)% |
| median no. (IQR) family members | 5 (IQR: 4-8) | 5 (IQR: 3-7) |
| No. of pedigrees with one or more family members affected with breast/ovarian cancer | 23 (47.9%) | 23 (54.8%) |
| No. of pedigrees with additional members (other than proband) tested for mutation | 12 (25.0%) | 15 (35.7%) |
| **Multiple-case families** | | |
| Total families | 46 (13.2%) | 32 (10.6%) |
| Mean (SD) age of diagnosis of breast/ovarian cancer of index cases | 40.9 (10.5) | 44.9 (12.9) |
| Ethnicity of index cases |  |  |
| Chinese | 27 (75.0%) | 26 (81.2%) |
| Malay | 5 (13.9%) | 3 (9.4%) |
| Indian | 4 (11.1%) | 3 (9.4%) |
| median no. (IQR) family members | 5 (IQR: 4-7) | 6 (IQR:4-7) |
| No. of pedigrees with one or more family members affected with breast/ovarian cancer | 24 (66.7%) | 19 (59.4%) |
| No. of pedigrees with additional members (other than proband) tested for mutation | 36 (100%) | 32 (100%) |

**eTable 4: Summary of disease status (first cancer) of family member by gene mutated of proband, recruitment country and age of diagnosis (or age of censoring)**

| **Age** | **BRCA1** | | | | | | **BRCA2** | | | | | | |
| --- | --- | --- | --- | --- | --- | --- | --- | --- | --- | --- | --- | --- | --- |
|  | **Malaysia** | | | **Singapore** | | | **Malaysia** | | | **Singapore** | | | |
|  | **UN** | **Breast** | **Ovarian** | **UN** | **Breast** | **Ovarian** | **UN** | **Breast** | **Ovarian** | **UN** | **Breast** | **Ovarian** |  |
| <20 | 72 | 0 | 1 | 19 | 0 | 0 | 52 | 0 | 0 | 38 | 0 | 0 |  |
| 20-29 | 83 | 4 | 1 | 42 | 2 | 0 | 60 | 3 | 0 | 60 | 0 | 0 |  |
| 30-39 | 102 | 28 | 3 | 55 | 10 | 2 | 89 | 17 | 0 | 55 | 4 | 0 |  |
| 40-49 | 93 | 23 | 7 | 44 | 24 | 9 | 104 | 27 | 2 | 68 | 33 | 1 |  |
| 50-59 | 108 | 17 | 17 | 58 | 21 | 6 | 130 | 20 | 0 | 102 | 27 | 3 |  |
| 60-69 | 77 | 5 | 10 | 66 | 5 | 6 | 113 | 7 | 6 | 89 | 9 | 3 |  |
| 70-79 | 65 | 3 | 1 | 28 | 2 | 2 | 89 | 3 | 1 | 55 | 2 | 3 |  |
| Total | 600 | 80 | 40 | 312 | 64 | 25 | 637 | 77 | 9 | 467 | 75 | 10 |  |

UN: unaffected

**eTable 6: Effect of country, birth cohort, ethnicity and PV location on breast and ovarian cancer risk of carriers of PVs in *BRCA1* and *BRCA2* from Malaysia and Singapore.**

|  | **RR (95% Floating CI^a^)** | | | | | | | |  |
| --- | --- | --- | --- | --- | --- | --- | --- | --- | --- |
|  | **BRCA 1 (N = 271)** | | | | **BRCA 2 (N = 301)** | | | |  |
|  | **No. Affected relative** | **Breast** | **No. Affected relative** | **Ovarian** | **No. Affected relative** | **Breast** | **No. Affected relative** | **Ovarian** |  |
| **Country** | | | | | | | | |  |
| **Malaysia** | 80 | 1 | 39 | 1 | 77 | 1 | 9 | 1 |  |
| **Singapore** | 64 | 0.7 | 25 | 0.8 | 75 | 0.8 | 10 | 1.1 |  |
|  |  | (0.5-1.1) |  | (0.5-1.5) |  | (0.5-1.2) |  | (0.3-3.4) |  |
| **Birth cohort** | | | | | | | | |  |
| **<1940** | 18 | 1  (0.5-2.0) | 7 | 1 (0.4-2.8) | 20 | 1  (0.5-2.0) | 4 | 1  (0.6-1.6) |  |
| **1940-1959** | 73 | 2.5 | 43 | 7.8 | 78 | 1.4 | 14 | 3.3 |  |
|  |  | (1.8-3.4) |  | (5.3-11.6) |  | (1.1-2.2) |  | (1.1-10.2) |  |
| **1960-1969** | 29 | 2.9 | 11 | 10.7 | 32 | 2.2 | 0 | - |  |
|  |  | (1.9-4.6) |  | (5.2-22.1) |  | (1.4-3.4) |  |  |  |
| **1970 onwards** | 24 | 3.3 | 3 | 5.7 | 22 | 4.4 | 1 | 23.1 |  |
|  |  | (1.9-5.7) |  | (1.1-30.5) |  | (2.3-8.5) |  | (2.1-256.2) |  |
| **Ethnicity** | | | | | | | | |  |
| **Indian** | 26 | 1  (0.6-1.6) | 6 | 1  (0.7-1.5) | 14 | 1  (0.5-1.9) | 5 | 1 |  |
| **Malay** | 30 | 0.6  (0.4-1.0) | 21 | 1.5  (0.9-2.5) | 38 | 2.3  (1.6-3.5) | 1 | - |  |
| **Chinese** | 88 | 0.7  (0.6-1.0) | 37 | 1.6  (0.9-2.5) | 100 | 1.1  (0.9-1.5) | 13 | 0.5  (0.2-1.4) |  |
|  |  |  |  |  |  |  |  |  |  |
| **Mutation location^b^** | | | | | | | | |  |
| **Group 1** | 53 | 1.5  (1.0-2.1) | 20 | 1.3  (0.8-2.2) | 38 | 0.7  (0.5-1.1) | 4 | 0.4  (0.1-1.7) |  |
| **Group 2** | 46 | 1.0  (0.7-1.4) | 29 | 1.0  (0.7-1.4) | 53 | 1  (0.7-1.4) | 6 | 1  (0.4-2.4) |  |
| **Group 3** | 45 | 1.4  (1.0-2.1) | 15 | 1.0  (0.6-1.8) | 61 | 0.8  (0.6-1.2) | 9 | 0.8  (0.3-1.9) |  |
| **Ascertainment adjustment** | | | | | | | | |  |
| **Unselected for FH** | 121 | 1 | 57 | 1 | 124 | 1 | 14 | 1 |  |
| **High risk Families** | 23 | 0.9 (0.3-3.3) | 7 | 1.2  (0.1-10.8) | 28 | 3.6  (0.8 - 15.6) | 5 | 14.2  (0.1-22.92) |  |

^a^Floating confidence intervals were reported for the subgroup analyses with more than two categories.

^b^*BRCA1*: Group 1 - 5′ to c.2281, Group 2 - c.2282 to c.4071, Group 3 - c.4072 to 3′; *BRCA2*: Group 1 - 5′ to c.2830, Group 2 – c.2831 to c.6401 (OCCR), Group 3 - c.6402 to 3′.

**eTable 7: Effect of birth cohort and ethnicity on breast and ovarian cancer risk of carriers of PV in *BRCA1* and *BRCA2* from the Malaysia studies only (see eTable 2)**

|  | **RR (95% Floating CI)** | | | | | | | | |
| --- | --- | --- | --- | --- | --- | --- | --- | --- | --- |
|  | **BRCA 1 (N = 156)** | | | | **BRCA 2 (N = 158)** | | | | |
|  | **No. Affected relative** | **Breast** | **No. Affected relative** | **Ovarian** | **No. Affected relative** | **Breast** | **No. Affected relative** | **Ovarian** |  |
| **Birth cohort** | | | | | | | | | |
| **<1940** | 11 | 1  (0.4-2.2) | 4 | 1  (0.3-3.4) | 12 | 1  (0.4-2.3) | 2 | 1  (0.5-2.1) |  |
| **1940-1959** | 35 | 1.8 | 29 | 8.3 | 37 | 1.6 | 6 | 3.2 |  |
|  |  | (1.2-2.7) |  | (5.1-13.5) |  | (1.1-2.4) |  | (0.8-12.7) |  |
| **1960-1969** | 18 | 2.6 | 4 | 7.6 | 14 | 2.4 | 0 | - |  |
|  |  | (1.5-4.5) |  | (2.5-23.0) |  | (1.3-4.4) |  |  |  |
| **1970 onwards** | 16 | 3.3 | 2 | 10 | 14 | 4.6 | 1 | 31.2 |  |
|  |  | (1.8-6.2) |  | (1.9-54.0) |  | (2.1-10.3) |  | (2.3-429.4) |  |
| **Ethnicity** | | | | | | | | | |
| **Indian** | 21 | 1  (0.6-1.9) | 3 | 1  (0.3-3.6) | 9 | 1  (0.5-2.2) | 3 | 1 |  |
| **Malay** | 21 | 0.7  (0.4-1.1) | 15 | 2.8  (1.5-5.0) | 29 | 2.5  (1.6-4.0) | 1 | - |  |
| **Chinese** | 38 | 0.9 (0.6-1.3) | 21 | 4  (2.4-6.9) | 39 | 1.0  (0.7-1.5) | 5 | 0.4 |  |
|  |  |  |  |  |  |  |  | (0.1-1.5) |  |

RR: relative risk; n: number of affected female relatives

**eTable 8: Estimated relative risk of breast and ovarian cancer for PV carriers of *BRCA1* or *BRCA2***

| **Model** | **BRCA 1** | | | |  | **BRCA 2** | | | |  |
| --- | --- | --- | --- | --- | --- | --- | --- | --- | --- | --- |
|  | **Southeast Asian** | | **Korean** | **European** | **p-value^a^** | **Southeast Asian** | | **Korean** | **European** | **p-value^a^** |
|  | **(N = 233)** | | **(N = 151)** | **(N = 280)** |  | **(N = 283)** | | **(N = 225)** | **(N = 218)** |  |
|  | **No. affected relatives** | **RR** | **RR** | **RR** |  | **No. affected relatives** | **RR** | **RR** | **RR** |  |
|  |  | **(95% CI)** | **(95% CI)** | **(95% CI)** |  |  | **(95% CI)** | **(95% CI)** | **(95% CI)** |  |
| **Breast cancer** | | | | | | | | | | |
| **Age constant** | | | | | | | | | | |
| **20-79** | 144 | 15.6 | 18 | - | 0.876 | 152 | 10.3 | 11 | - | 0.874 |
|  |  | (12.6-19.4) | (3.0–103.0) |  |  |  | (8.3-12.7) | (5.0–27.0) |  |  |
| **Age stratified** | | | | | | | | | | |
| **20-29** | 6 | 25.1 | - | 18 | 0.71 | 3 | 9.7 | - | 19 | 0.538 |
|  |  | (9.1-69.3) |  | (4.4-75) |  |  | (2-46.1) |  | (4.4-82) |  |
| **30-39** | 38 | 27 | - | 36 | 0.27 | 21 | 9.5 | - | 16 | 0.177 |
|  |  | (18.9-38.9) |  | (25-52) |  |  | (5.6-16.1) |  | (9.3 – 29.0) |  |
| **40-49** | 47 | 16.1 | - | 31 | **0.039** | 60 | 14.1 | - | 9.5 | 0.175 |
|  |  | (11.4-22.8) |  | (25-52) |  |  | (10.3-19.4) |  | (5.9-15) |  |
| **50-59** | 38 | 12.1 | - | 16 | 0.416 | 47 | 10.9 | - | 11 | 0.977 |
|  |  | (7.9-18.5) |  | (9.6-27) |  |  | (7.6-15.6) |  | (6.6-17) |  |
| **60-69** | 10 | 6.4 | - | 11 | 0.366 | 16 | 4.8 | - | 9.2 | 0.167 |
|  |  | (2.8-14.8) |  | (5-25) |  |  | (2.4-9.8) |  | (5.1-17) |  |
| **70-79** | 5 | 10.1 | - | - | - | 5 | 4.9 | - | - | **-** |
|  |  | (3.4-30.0) |  |  |  |  | (1.5-15.7) |  |  |  |
| **Ovarian cancer** | | | | | | | | | | |
| **Age constant** | | | | | | | | | | |
| **20-79** | 64 | 39.8 | - | - | - | 19 | 6.8 | - | - | - |
|  |  | (29.6-53.3) |  |  |  |  | (3.9-11.9) |  |  |  |
| **Age stratified** | | | | | | | | | | |
| **20-29** | 1 | 8.7 | - | - | - | 0 | - | - | - | - |
|  |  | (0.9-79.4) |  |  |  |  |  |  |  |  |
| **30-39** | 5 | 10.3 | - | 38 | 0.08 | 0 | - | - | - | - |
|  |  | (3-35.1) |  | (17-88) |  |  |  |  |  |  |
| **40-49** | 16 | 35.5 | - | 61 | 0.142 | 3 | 2.3 | - | 6.3 | 0.437 |
|  |  | (20.5-61.4) |  | (38-99) |  |  | (0.3-17.7) |  | (1.4-28) |  |
| **50-59** | 23 | 51.9 | - | 30 | 0.243 | 3 | 6.3 | - | 19 | 0.098 |
|  |  | (32.0-84.2) |  | (14-65) |  |  | (2.2-18.6) |  | (9.1-41) |  |
| **60-69** | 16 | 81.7 | - | 48 | 0.274 | 9 | 15.8 | - | 7.3 | 0.363 |
|  |  | (47.0-141.9) |  | (22-109) |  |  | (6.4-38.9) |  | (1.8-30) |  |
| **70-79** | 3 | 25.6 | - | - | - | 4 | 29.5 | - | - | **-** |
|  |  | (5.5-119.5) |  |  |  |  | (10.1-86.2) |  |  |  |

^a^z-test for comparison of RRs between two populations.

**eTable 9: Breast and ovarian cancer incidences and cumulative risks of carriers of mutations in BRCA1 and BRCA 2 (Singapore)**

| **Ages** | **Incidence per 1000 Person-years (95% CI)** | | | | **Cumulative risk, % (95% CI)** | | | |
| --- | --- | --- | --- | --- | --- | --- | --- | --- |
|  | **Breast^a^** | | | **Ovarian^b^** | **Breast^a^** | | | **Ovarian^b^** |
|  | **Chinese** | **Malay** | **Indian** |  | **Chinese** | **Malay** | **Indian** |  |
| ***BRCA1* mutation** | | | | | | | | |
| **20** | 0 | 0 | 0 | 0 | 0 | 0 | 0 | 0 |
| **25** | 0 | 0 | 0 | 0 | 0 | 0 | 0 | 0 |
| **30** | 3 (2-5) | 4 (2-5) | 2 (1-3) | 0 | 1 (1-1) | 1 (1-2) | 0 (0-1) | 0 |
| **35** | 9 (6-13) | 8 (6-12) | 5 (3-7) | 1 (0-4) | 4 (4-5) | 4 (4-5) | 2 (2-3) | 1 (0-1) |
| **40** | 14 (10-20) | 13 (9-18) | 9 (6-13) | 3 (2-6) | 10 (8-11) | 9 (8-11) | 6 (5-7) | 2 (1-3) |
| **45** | 20 (14-29) | 18 (13-26) | 15 (10-21) | 8 (5-13) | 17 (15-20) | 16 (15-18) | 11 (10-13) | 5 (3-6) |
| **50** | 24 (15-36) | 21 (14-33) | 19 (12-29) | 10 (6-17) | 26 (24-29) | 24 (22-27) | 19 (17-21) | 9 (7-11) |
| **55** | 25 (16-39) | 22 (15-34) | 22 (14-34) | 13 (8-21) | 35 (32-38) | 32 (29-36) | 27 (24-30) | 14 (13-16) |
| **60** | 24 (11-57) | 22 (9-50) | 23 (10-54) | 14 (8-24) | 42 (39-46) | 39 (36-43) | 35 (31-39) | 20 (18-22) |
| **65** | 22 (10-52) | 20 (8-45) | 23 (10-53) | 15 (9-26) | 49 (45-53) | 45 (41-49) | 42 (37-46) | 26 (22-29) |
| **70** | 20 (7-59) | 17 (6-51) | 21 (7-63) | 16 (4-71) | 54 (49-58) | 50 (46-54) | 48 (43-53) | 31 (26-37) |
| **75** | 17 (6-50) | 14 (5-42) | 19 (6-57) | 17 (4-73) | 58 (53-62) | 54 (49-58) | 53 (48-58) | 37 (30-44) |
| **80** | 14 (5-41) | 11 (4-34) | 17 (6-49) | 17 (4-74) | 61 (56-66) | 57 (52-61) | 57 (51-63) | 42 (34-50) |
| ***BRCA2* mutation** | | |  |  |  |  |  |  |
| **20** | 0 | 0 | 0 | 0 | 0 | 0 | 0 | 0 |
| **25** | 0 | 0 | 0 | 0 | 0 | 0 | 0 | 0 |
| **30** | 1 (1-2) | 2 (1-3) | 1 (0-1) | 0 | 0 (0-1) | 0 (0-1) | 0 (0-0) | 0 |
| **35** | 4 (2-7) | 4 (2-7) | 2 (1-4) | 0 | 2 (2-2) | 2 (2-2) | 1 (1-1) | 0 |
| **40** | 7 (5-10) | 7 (5-9) | 5 (4-7) | 0 | 5 (4-6) | 5 (4-5) | 3 (3-3) | 0 |
| **45** | 12 (9-17) | 11 (8-15) | 9 (7-12) | 1 (0-4) | 10 (8-11) | 9 (8-10) | 6 (5-7) | 0 |
| **50** | 17 (12-24) | 15 (11-22) | 14 (9-20) | 1 (0-3) | 16 (14-18) | 15 (13-17) | 12 (10-13) | 1 (0-1) |
| **55** | 21 (15-30) | 19 (13-27) | 18 (13-26) | 2 (1-5) | 24 (22-27) | 22 (20-25) | 19 (17-21) | 1 (1-2) |
| **60** | 19 (9-38) | 17 (8-34) | 18 (9-36) | 3 (1-7) | 31 (28-34) | 29 (26-31) | 26 (23-29) | 2 (2-3) |
| **65** | 16 (8-32) | 14 (7-28) | 16 (8-32) | 5 (2-12) | 37 (34-40) | 34 (31-37) | 32 (29-35) | 4 (3-6) |
| **70** | 13 (4-41) | 11 (3-35) | 14 (4-44) | 8 (3-22) | 41 (38-45) | 38 (35-41) | 36 (33-40) | 7 (5-12) |
| **75** | 10 (3-32) | 8 (3-27) | 11 (3-36) | 13 (5-36) | 44 (41-48) | 41 (37-44) | 40 (36-45) | 12 (8-18) |
| **80** | 7 (2-24) | 6 (2-19) | 9 (3-29) | 21 (8-56) | 47 (43-51) | 43 (39-46) | 43 (39-48) | 20 (14-27) |

^a^Incidences and cumulative risks for breast cancer were calculated based on population calendar, cohort and ethnic-specific breast cancer incidences for a woman born in Singapore between 1950 and 1959. Mortality was not accounted for in cumulative risk estimates.

^b^Incidences and cumulative risks for ovarian cancer were calculated based on overall population calendar and cohort-specific ovarian cancer incidences for a woman born in Singapore between 1950 and 1959, as ethnic-specific ovarian cancer incidence was not available. Mortality was not accounted for in cumulative risk estimates.

**eFigure 1: Lollipop plot of probands with (A) *BRCA1* and (B) *BRCA2* mutations using cBioPortal (v4.1.9).** Probands with breast cancer (BC) are shown in the top panel and probands with ovarian cancer (OC) are shown in the bottom panel. Probands with both breast and ovarian cancer (BC+OC) are included in both top and bottom panels. Position of large genomic rearrangements are listed below the lollipop plot with the length of exon deletions or duplications (coloured bars). This plot excludes 2 *BRCA1* probands and 7 *BRCA2* probands with no personal or family history of breast or ovarian cancer, and 1 *BRCA1* proband with no variant information.


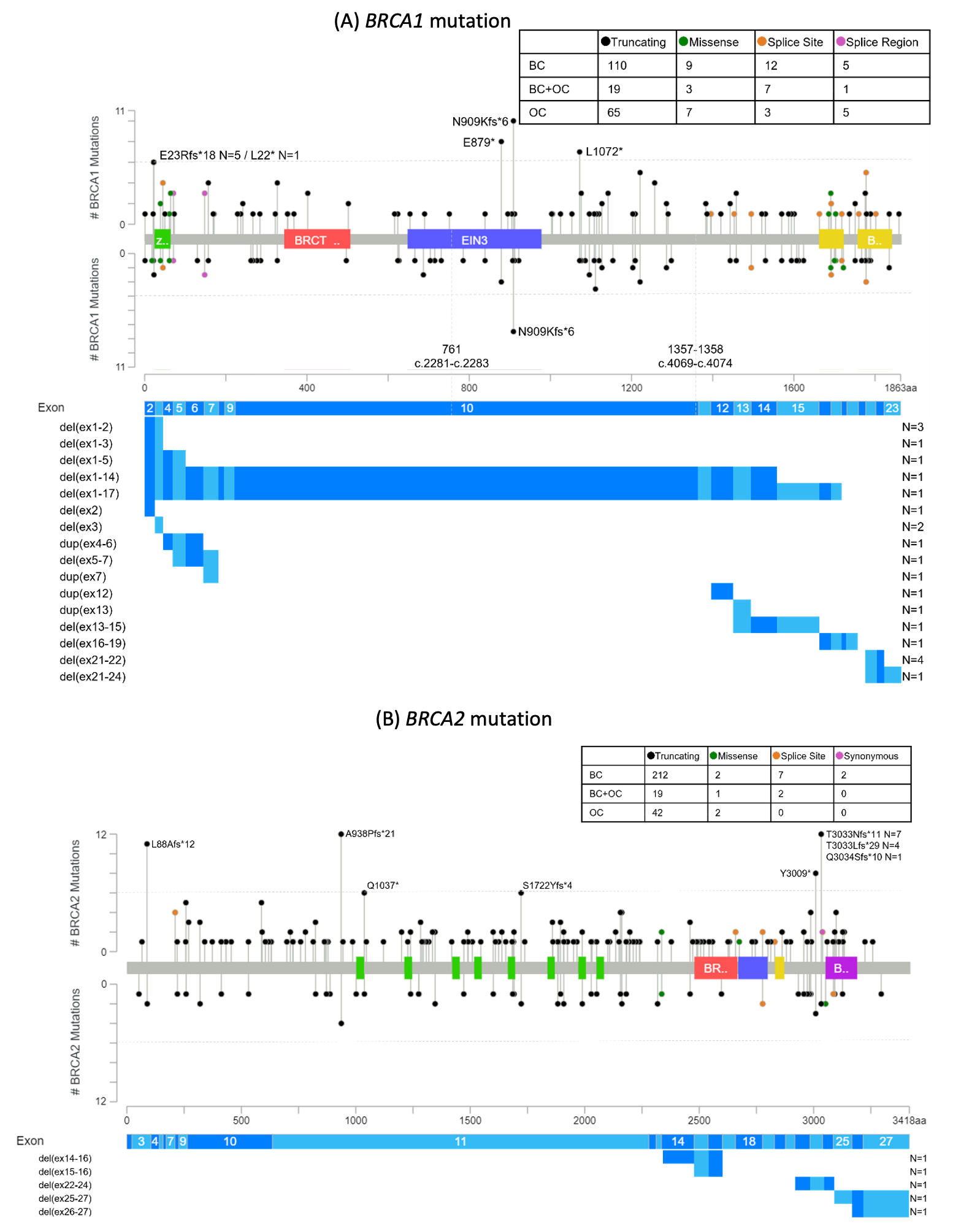


**eFigure 2: Principal components plot of index cases of self-reported Chinese (red dots), Malay (blue dots) and Indian (black dots) from MyBrCa, SGBCC, MyOvCa, MaGiC and MyF studies.**

**Supplementary files description**

Supplementary file (eTable 5) gives the list of pathogenic and likely pathogenic variants identified in the study.

**Consortia membership**

*SGBCC investigators:*

Benita Kiat-Tee Tan, Su-Ming Tan, Veronique Kiak Mien Tan, Ern Yu Tan, Geok Hoon Lim, Alexis Khng

*MaGiC investigators:*

Gaik‑Siew Ch’ng, Jamil Omar, Chee‑Meng Yong, Ismail Aliyas, Rozita Abdul Malik, Suguna Subramaniam, Wee‑Wee Sim, Chun‑Sen Lim, Saw‑Joo Lee, Keng‑Joo Lim, Mohamad Nasir Shafiee, Fuad Ismail, Mohd Pazudin Ismail, Mohamad Faiz Mohamed Jamli, Suresh Kumarasamy, John S. H. Low, Ahmad Muzamir Ahmad Mustafa, Mary J. Makanjang, Shahila Taib, Nellie L.C. Cheah, Chee‑Kin Fong, Kean‑Fatt Ho, Azura Deniel, Soo Fan Ang, Ahmad Radzi Ahmad Badruddin, Lye-Mun Tho

**References**

1. Tan M-M, Ho W-K, Yoon S-Y, et al. A case-control study of breast cancer risk factors in 7,663 women in Malaysia. *PloS one*. 2018;13(9):e0203469.

2. Ho PJ, Yeoh YS, Miao H, et al. Cohort profile: The Singapore Breast Cancer Cohort (SGBCC), a multi-center breast cancer cohort for evaluation of phenotypic risk factors and genetic markers. *PLoS One*. 2021;16(4):e0250102.

3. Hasmad HN, Lai KN, Wen WX, et al. Evaluation of germline BRCA1 and BRCA2 mutations in a multi-ethnic Asian cohort of ovarian cancer patients. *Gynecologic Oncology*. 2016;141(2):318-322.

4. Yoon S-Y, Wong SW, Lim J, et al. Oncologist-led BRCA counselling improves access to cancer genetic testing in middle-income Asian country, with no significant impact on psychosocial outcomes. *Journal of medical genetics*. 2022;59(3):220-229.

5. Amos CI, Dennis J, Wang Z, et al. The OncoArray Consortium: A Network for Understanding the Genetic Architecture of Common CancersThe OncoArray and Common Cancer Etiology. *Cancer epidemiology, biomarkers & prevention*. 2017;26(1):126-135.

6. Dorling L, Carvalho S, Allen J. Breast cancer risk genes—association analysis in more than 113,000 women. *New England Journal of Medicine*. 2021;384(5):428-439.

7. Wen WX, Allen J, Lai KN, et al. Inherited mutations in BRCA1 and BRCA2 in an unselected multiethnic cohort of Asian patients with breast cancer and healthy controls from Malaysia. *Journal of medical genetics*. 2018;55(2):97-103.

8. Lai Z, Markovets A, Ahdesmaki M, et al. VarDict: a novel and versatile variant caller for next-generation sequencing in cancer research. *Nucleic acids research*. 2016;44(11):e108-e108.

9. Yoon S, Bashah NA, Wong S, et al. Mainstreaming genetic counselling for genetic testing of BRCA1 and BRCA2 in ovarian cancer patients in Malaysia (MaGiC study). *Annals of Oncology*. 2017;28:x187.

10. Antoniou A, Pharoah PD, Narod S, et al. Average risks of breast and ovarian cancer associated with BRCA1 or BRCA2 mutations detected in case series unselected for family history: a combined analysis of 22 studies. *The American Journal of Human Genetics*. 2003;72(5):1117-1130.

11. Milne RL, Osorio A, Cajal TRny, et al. The average cumulative risks of breast and ovarian cancer for carriers of mutations in BRCA1 and BRCA2 attending genetic counseling units in Spain. *Clinical Cancer Research*. 2008;14(9):2861-2869.

12. Yang X, Leslie G, Doroszuk A, et al. Cancer risks associated with germline PALB2 pathogenic variants: an international study of 524 families. *Journal of clinical oncology*. 2020;38(7):674.

13. Antoniou AC, Cunningham A, Peto J, et al. The BOADICEA model of genetic susceptibility to breast and ovarian cancers: updates and extensions. *British journal of cancer*. 2008;98(8):1457-1466.

14. Easton DF, Peto J, Babiker AG. Floating absolute risk: an alternative to relative risk in survival and case‐control analysis avoiding an arbitrary reference group. *Statistics in medicine*. 1991;10(7):1025-1035.

15. Kuchenbaecker KB, Hopper JL, Barnes DR, et al. Risks of breast, ovarian, and contralateral breast cancer for BRCA1 and BRCA2 mutation carriers. *Jama*. 2017;317(23):2402-2416.

16. Singapore Cancer Registry. 50 years of registration: Singapore Cancer Registry. 2019.

17. Zarihah M, Mohd Yusoff H, Devaraj T. Penang Cancer Registry Report 1994-1998. *Penang: Penang Cancer Registry*. 2003;

18. Bina Rai S, Devaraj T, Aishah K, Rokiah M, Nor Asikin A. Penang Cancer Registry Report 1999–2003. *Penang, Penang Cancer Registry*. 2005;

19. Azizah A, Devaraj T, Bina R, Yusuff N, Mansoor N, Shuib N. Penang Cancer Registry Report 2004–2008. *Penang Cancer registry*. 2010;

20. Azizah A, Hashimah B, Nirmal K, et al. Malaysia National cancer registry report (MNCR). 2019;

21. Delon C, Brown KF, Payne NW, Kotrotsios Y, Vernon S, Shelton J. Differences in cancer incidence by broad ethnic group in England, 2013–2017. *British Journal of Cancer*. 2022:1-9.

22. Park B, Dowty JG, Ahn C, et al. Breast cancer risk for Korean women with germline mutations in BRCA1 and BRCA2. *Breast cancer research and treatment*. 2015;152(3):659-665.

23. Yao L, Sun J, Zhang J, et al. Breast cancer risk in Chinese women with BRCA1 or BRCA2 mutations. *Breast cancer research and treatment*. 2016;156(3):441-445.

24. Zhang L, Shin VY, Chai X, et al. Breast and ovarian cancer penetrance of BRCA1/2 mutations among Hong Kong women. *Oncotarget*. 2018;9(38):25025.

25. Momozawa Y, Sasai R, Usui Y, et al. Expansion of Cancer Risk Profile for BRCA1 and BRCA2 Pathogenic Variants. *JAMA oncology*. 2022;
