# Supplementary material for: Age-specific breast and ovarian cancer risks associated with germline BRCA1 or BRCA2 pathogenic variants – an Asian study of 572 families
Source: Lancet Reg Health West Pac. 2024 Feb 5;44:101017. doi: 10.1016/j.lanwpc.2024.101017 (PMC10851205; doi:10.1016/j.lanwpc.2024.101017)
Supplement: Supplementary material_Names of Investigators within MaGiC and SGBCC [file mmc3.docx]

*MaGiC Investigators*

| First & Middle Name | Last Name |  |
| --- | --- | --- |
| Gaik‑Siew | Ch’ng |  |
| Jamil | Omar |  |
| Chee‑Meng | Yong |  |
| Ismail | Aliyas |  |
| Rozita | Abdul Malik |  |
| Suguna | Subramaniam |  |
| Wee‑Wee | Sim |  |
| Chun Sen | Lim |  |
| Saw‑Joo | Lee |  |
| Keng‑Joo | Lim |  |
| Mohamad Nasir | Shafiee |  |
| Fuad Ismail | Ismail |  |
| Mohd Pazudin | Ismail |  |
| Mohamad Faiz | Mohamed Jamli |  |
| Suresh | Kumarasamy |  |
| John S. H | Low |  |
| Ahmad Muzamir | Ahmad Mustafa |  |
| Mary J. | Makanjang |  |
| Shahila | Taib |  |
| Nellie | Cheah |  |
| Chee‑Kin | Fong |  |
| Kean‑Fatt | Ho |  |
| Azura | Deniel |  |
| Soo Fan | Ang |  |
| Ahmad Radzi | Ahmad Badruddin |  |
| Lye-Mun | Tho |  |
|  | |  |
| *SGBCC Investigators* | |  |
|  |  |  |
| First & Middle Name | | Last Name |
| Benita Kiat-Tee | | Tan |
| Su-Ming | | Tan |
| Veronique Kiak Mien | | Tan |
| Ern Yu | | Tan |
| Geok Hoon | | Lim |
| Alexis | | Khng |
